# Genetic Screening to Identify Candidate Resistance Alleles to Cry1F Corn in Fall Armyworm Using Targeted Sequencing

**DOI:** 10.3390/insects12070618

**Published:** 2021-07-08

**Authors:** Katrina Schlum, Kurt Lamour, Peter Tandy, Scott J. Emrich, Caroline Placidi de Bortoli, Tejas Rao, Diego M. Viteri Dillon, Angela M. Linares-Ramirez, Juan Luis Jurat-Fuentes

**Affiliations:** 1 Genome Science and Technology Graduate Program, University of Tennessee, Knoxville, TN 37996, USA; kschlum@vols.utk.edu (K.S.); klamour@utk.edu (K.L.); semrich@utk.edu (S.J.E.); 2 Department of Entomology and Plant Pathology, University of Tennessee, Knoxville, TN 37996, USA; ptandy@vols.utk.edu (P.T.); cplacidi@utk.edu (C.P.d.B.); trao2@utk.edu (T.R.); 3 Department of Electrical Engineering and Computer Science, University of Tennessee, Knoxville, TN 37996, USA; 4 Isabela Research Substation, Department of Agro-Environmental Sciences, University of Puerto Rico, Isabela, PR 00662, USA; diego.viteri@upr.edu; 5 Lajas Research Substation, Department of Agro-Environmental Sciences, University of Puerto Rico, Lajas, PR 00667, USA; angela.linares@upr.edu

**Keywords:** *Spodoptera frugiperda*, fall armyworm, resistance, Cry1F, genotyping, targeted sequencing, resistance screen, ABCC2

## Abstract

**Simple Summary:**

Monitoring of resistance alleles is critical to the sustainability of transgenic crops producing insecticidal Cry proteins. Highly sensitive and cost-effective DNA-based methods are needed to improve current bioassay-based resistance screening. Our goal was to evaluate the use of targeted sequencing in detecting known and novel candidate resistance alleles to Cry proteins. As a model, we used field-collected fall armyworm (*Spodoptera frugiperda*) from Puerto Rico, the first location reporting continued practical field-evolved *S. frugiperda* resistance to corn producing the Cry1F insecticidal protein, and sequenced the *SfABCC2* gene previously identified as critical to Cry1F toxicity. Targeted sequencing of *SfABCC2* detected a previously reported Cry1F resistance allele and mutations originally identified in populations from Brazil. Importantly, targeted sequencing also identified nonsynonymous and frameshift mutations as novel candidate resistance alleles. These results advocate for the use of targeted sequencing in screening for resistance alleles to Cry proteins and support potential gene flow, including resistance alleles, between *S. frugiperda* from Brazil and the Caribbean.

**Abstract:**

Evolution of practical resistance is the main threat to the sustainability of transgenic crops producing insecticidal proteins from *Bacillus thuringiensis* (Bt crops). Monitoring of resistance to Cry and Vip3A proteins produced by Bt crops is critical to mitigate the development of resistance. Currently, Cry/Vip3A resistance allele monitoring is based on bioassays with larvae from inbreeding field-collected moths. As an alternative, DNA-based monitoring tools should increase sensitivity and reduce overall costs compared to bioassay-based screening methods. Here, we evaluated targeted sequencing as a method allowing detection of known and novel candidate resistance alleles to Cry proteins. As a model, we sequenced a Cry1F receptor gene (*SfABCC2*) in fall armyworm (*Spodoptera frugiperda*) moths from Puerto Rico, a location reporting continued practical field resistance to Cry1F-producing corn. Targeted sequencing detected a previously reported Cry1F resistance allele (*SfABCC2mut*), in addition to a resistance allele originally described in *S. frugiperda* populations from Brazil. Moreover, targeted sequencing detected mutations in *SfABCC2* as novel candidate resistance alleles. These results support further development of targeted sequencing for monitoring resistance to Bt crops and provide unexpected evidence for common resistance alleles in *S. frugiperda* from Brazil and Puerto Rico.

## 1. Introduction

The fall armyworm (*Spodoptera frugiperda*, J.E. Smith) (Lepidoptera: Noctuidae) is a global invasive pest affecting numerous food and fiber staple crops, although the highest damage is observed in corn (*Zea mays* L.) [1]. In North America, *S. frugiperda* moths display long-distance northward migration over several generations from overwintering sites in southern Texas and Florida, reaching Canada in the summer months [2]. Substantial gene flow also occurs between *S. frugiperda* populations from the Caribbean and Florida, but not between these populations and moths from South America [3]. 

Effective control of *S. frugiperda* in the Western hemisphere in addition to pesticides, has been provided by genetically modified corn and cotton producing insecticidal proteins from the bacterium *Bacillus thuringiensis* (*Bt*), namely Cry1F, Cry1Ab, Cry1A.105, Cry2Ab and Vip3Aa. In the past decade, resistance to Cry1F, Cry1Ab and Cry1A.105 was reported for *S. frugiperda* populations in Puerto Rico and the continental US (Florida and North Carolina) [4,5], Brazil [6] and Argentina [7]. Fall armyworm resistance to these Cry proteins is recessive and genetically linked to mutations in an ABC transporter superfamily C2 gene (*SfABCC2*) [8,9,10]. In Puerto Rico, two *SfABCC2* resistance alleles have been reported, a nonsense 2 bp insertion (*SfABCC2mut* allele) [8] and an insertion near the start of the fourth exon associated with aberrant splicing [9]. In contrast, resistance to Cry1F in Brazil is linked to missense mutations primarily localized to the fourth extracellular loop in SfABCC2, in support of this region containing a Cry1F binding domain [11] and being a hotspot for resistance mutations [10]. Genetic knockout of *SfABCC2* leads to resistance against Cry1F [12,13] further supporting that truncation of SfABCC2 is predictive of a Cry1F-resistant phenotype.

Identification of resistance alleles in *S. frugiperda* is critical to developing highly sensitive DNA-based genotyping tools, which are less laborious and costly compared to F1 [14] or F2 [5] resistance screens. Successful *SfABCC2* genotyping efforts were described using TaqMan probes [8,9] and pyrosequencing [10] in populations from Puerto Rico, USA and Brazil. Results from these tests support high-resistance allele frequencies in areas where practical resistance had been reported, further associating identified *SfABCC2* alleles with field-relevant resistance. An unexpected observation, considering predicted migratory models [3], was not detecting the *SfABCC2mut* allele reported in Puerto Rico at migratory Caribbean destinations or in the Florida populations tested [8,9]. Relevant fitness costs are not associated with *SfABCC2mut* under laboratory conditions [15] and thus probably do not explain the limited distribution to Puerto Rico of *SfABCC2mut*. 

Hi-Plex (HP) is a highly multiplexed PCR-based approach for targeted massive parallel sequencing with demonstrated efficacy in disease gene screening [16,17]. It involves amplifying small (e.g., 180 bp) overlapping portions of a larger target region (tiling) in one (or a few) multiplex PCR reaction(s) adding a unique sample-specific barcode sequence during the PCR reaction. The HP technology is attractive because it requires very little genomic DNA to amplify multiple target amplicons (e.g., 20–40 ng of template DNA to amplify a few hundred targets), the DNA can be highly complex (e.g., mixed with genomic DNA from many different organisms) and the overall cost in time and reagents is greatly reduced compared to F1 and F2 screens. The barcoded amplicons are pooled and sequenced on a next-generation sequencing (NGS) device, and the sample-specific amplicons mapped to a reference genome. 

The goal of this project was to adapt and provide preliminary data for a new HP targeted screen for resistance alleles to *Bt* insecticidal proteins. We focused our analysis on *S. frugiperda* samples from Puerto Rico, as both *SfABCC2mut* and Cry1F resistance are highly frequent at this location [8,18]. Furthermore, mutations resulting in a truncated SfABCC2 protein result in a Cry1F-resistant phenotype [8,9,12,13], potentially allowing for phenotypic predictions. Analysis of Taqman and targeted *SfABCC2* sequences detected the known *SfABCC2mut* allele in 34–37% of tested samples, depending on the technique used. In addition, the HP approach coupled to a bioinformatics pipeline detected one nonsense and nine frameshift *SfABCC2* mutations as novel candidate Cry1F resistance alleles. Missense mutations (13) were also identified, but phenotype predictions are not possible in this case as the effect of these mutations on Cry1F susceptibility and resistance requires functional testing. We document, for the first time, that a *SfABCC2* resistance allele previously described in *S. frugiperda* from Brazil is present in Puerto Rico fall armyworm populations. These data provide proof of concept for the power of HP targeted sequencing for performing surveillance and field monitoring of well-established and novel candidate *Bt* resistance alleles. 

## 2. Materials and Methods

### 2.1. S. frugiperda Sample Collection and Genomic DNA Extraction

Adult moths were captured at three locations in Puerto Rico (Lajas, Salinas and Santa Isabel) using sex pheromone-baited traps [19] at sites near field and sweet/field corn plantings in order to optimize the capture efficiency of C-strain males. Collection details of the 41 *S. frugiperda* individuals tested are in Table S1. Captured moths were confirmed as *S. frugiperda* by visual inspection [20] and kept refrigerated until shipped to the University of Tennessee. Samples were stored at −20 °C until processed for purification of genomic DNA. Samples were named and numbered according to trapping location as PRL (Lajas), PRS (Salinas) and PRSI (Santa Isabel). A sample from the previously characterized Cry1F-resistant 456LSD4 strain of *S. frugiperda*, which originated from collections in Puerto Rico [21], was also included as positive control for detection of *SfABCC2mut* [8].

Genomic DNA was isolated from moth legs using the Pure Link Genomic DNA mini kit (Invitrogen) following manufacturer’s protocols, and then quantified using a Nanodrop spectrophotometer (Thermo Scientific). 

### 2.2. TaqMan Genotyping for SfABCC2mut 

Detection of the *SfABCC2mut* allele using Taqman probes was performed as previously described [8]. Briefly, 10 µL (final volume) reactions included 10–20 ng of gDNA as template, a VIC-labeled probe specific to the *SfABCC2mut* allele (5′ AAGCACATCGCCCACTT 3′), a FAM-labeled probe specific to the wild-type *SfABCC2* allele (5′ CCAAGCACATCCCACTT 3′), and forward (5′ TGGAGGCCGAAGAGAGACA 3′) and reverse (5′ AGGAGTTGACTGACTTCATGTACCT 3′) primers. Plates (Micro Amp Fast optical 96 well reaction plate, Applied Biosystems) were loaded in the Quant studio 6 Real Time PCR instrument (Applied Biosystems) and amplified as follows: pre read stage at 60 °C for 30 s, hold stage at 95 °C for 10 min, PCR stage at 95 °C for 15 s and 60 °C for 1 min for 40 cycles, post read stage at 60 °C for 30 s. The fluorescence in each well was measured in the post read stage of the PCR, and the software generated an allelic discrimination plot based on the post amplification intensity of the fluorescent probes.

The frequency of the *SfABCC2mut1* allele or any mutation was determined using the Hardy–Weinberg equation with the formula: F = (2 × ObsAa + Obsaa)/[2 × (ObsAA + ObsAa + Obsaa)], where “F” is the frequency of the “a” allele (*SfABCC2mut1*) and “Obs” the observed frequency of each of the three possible genotypes for the allele.

### 2.3. Targeted DNA Amplification and Sequencing

In early BLASTn searches of the *S. frugiperda* corn strain v3.1 genome from LepidoDB (https://bipaa.genouest.org/sp/spodoptera_frugiperda_pub/, accessed on 11 July 2019), we found that the *SfABCC2* gene was split between Scaffolds 11087 and 7154. These scaffolds were manually combined and the resulting *SfABCC2* gene sequence was used as a template to design TILING primers, using PCRTiler [22] at default settings allowing 50 bp overlap on each end and producing 180–200 bp final amplicon sizes (Table S2). The two approximately 1 Kbp intronic regions at the beginning of that gene sequence (Scaffold_11087) were not targeted. In a later version of the *S. frugiperda* corn strain genome (v6.0) from LepidoDB (accessed on 16 November 2020), the full-length *SfABCC2* gene appears in Scaffold 33. The initial *SfABCC2* gene assembly based on Scaffolds 11087 and 7154 used for TILING primer design did not include an inter-scaffold gap encompassing nucleotides 7239–8976 of the *SfABCC2* gene assembly in the v6.0 genome. The TILING primers covered approximately 90% of the exonic regions of the assembled *SfABCC2* gene found in Scaffold 33 of the *S. frugiperda* corn strain v6.0 genome (see Figure S1 for a visual representation of typical coverage).

All TILING primers (238) were mixed into a single multiplex (119 overlapping targets) and PCR performed using the Hi-Plex targeted sequencing strategy, as previously described [17]. The resulting amplicons were pooled and sequenced on a HiSeqX device running a 2 × 150 bp configuration and merged to produce single, high quality reads using PEAR [23]. Demultiplexing was accomplished using the sample-specific barcode sequences incorporated during the Hi-Plex PCR amplification.

### 2.4. Genotype Assignment for SfABCC2mut Using a Targeted-Sequencing k-mer-Based Approach

To genotype the *SfABCC2mut* 2 bp insertion in the *SfABCC2* targeted sequencing data, a custom Python 3 script (dubbed Pgeno) was developed and is deposited in Github at https://github.com/petertandy/pgeno (accessed on 26 May 2021). This script scans each of the sequence reads for the known mutation by using 10 mer flanking sequences as a guide. To account for unknown variation in the flanking regions, each of the 10 mers was allowed up to two mismatches. Sequences with matching flanks were counted and samples assigned a *SfABCC2mut* genotype based on the proportion of the known 2 bp insertions. In order to reduce false-positive genotype assignment, we required at least 40X sequence coverage in the PCR targeted regions and >15% of the total number of matching reads to assign a genotype of hetero- or homozygous for the 2 bp insertion. The minimum sequence coverage refers to the number of high-quality reads covering a target region in the *SfABCC2* reference gene and depends on the efficiency of the primers in the multiplex. The primer efficiencies are highly reproducible and by over-sequencing the samples, most of the gene is covered at a high depth (up to 1000X for some targets). These parameters can be adjusted in the Pgeno script to increase or decrease the genotyping stringency. 

### 2.5. Alignment, Variant Calling and Variant Analysis

**Figure 1:**
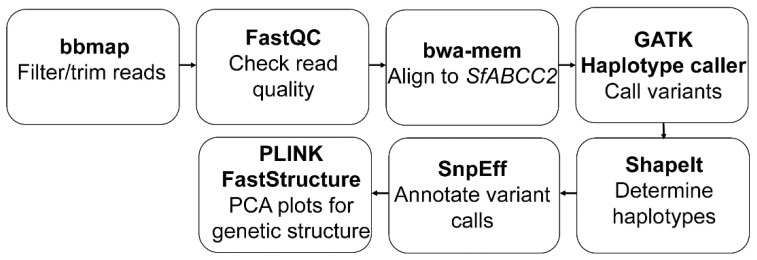
Schematic pipeline of overall workflow used in this work from raw Fastq targeted sequencing reads to population genetics and phylogenetic analyses based on bi-allelic SNP variant calling.

The complete pipeline used for trimming, alignment, variant calling, and analysis using targeted *SfABCC2* sequences is summarized in Figure 1. Raw reads were quality trimmed at both ends and filtered for adapter sequences using BBDuk [24]. The quality of samples was checked before and after trimming/processing to confirm adapter and nonrelevant (e.g., contaminants, primer artifacts) sequences were removed using FastQC [25]. The full-length *SfABCC2* gene interval was determined by aligning a *SfABCC2* cDNA from a Cry1F-susceptible *S. frugiperda* strain (GenBank accession number KY489760.1) to the *S. frugiperda* corn reference genome v6.0 [26] via the BLAST server hosted on LepidoDB. The full-length *SfABCC2* gene sequence was found included in Scaffold 33, and this reference gene sequence was used for further analyses. 

Reads were aligned to the *SfABCC2* reference gene using a Burrows–Wheeler aligner algorithm (bwa-mem) with default options [27]. The mapping rate for all samples was determined via SAMtools flagstat [28]. Only samples with a mapping rate of 80% or higher were kept, which led to the removal of only three samples from further analysis. We determined the count of DNA sequence reads across the *SfABCC2* gene using bam-readcount (https://github.com/genome/bam-readcount, accessed on 20 June 2019). As noted in 2.3, two regions (nucleotides ~138–1518 and ~7195–7858) within the *SfABCC2* gene were not sequenced due to the TILING primers being designed based on an old assembly (Scaffolds 11,087 and 7154 from *S. frugiperda* corn genome v3.1) of the gene. The second region representing the inter-scaffold gap in the early gene assembly was filled with ‘Ns’, precluding alignment of any reads to this region.

We performed alignment cleanup by sorting the bam file via SAMtools [28], deduplicating via Genome Analysis Tool Kit (GATK) version 4.1.20 [29] with MarkDuplicates and then adding read groups to each bam file via GATK’s function addOrReplaceReadGroup. Variants were called via the Haplotypecaller of GATK with default parameters set based on the GATK Best Practice pipeline. The initial set included 2006 variants with a quality score greater than 40. After considering different filters based on depth and minor allele frequencies, the final filter applied included depth of coverage ≥ 8, each individual allele depth ≥2, alternate allele frequency ≥5%, and the allele being called in at least 25% of the samples (>10). This filter produced 1861 variants (1428 SNPs and 433 indels), over 92% of the unfiltered set. We further filtered this variant set to include only bi-allelic variants using the GATK SelectVariants function, which resulted in 1333 bi-allelic variants (1143 SNPs and 190 indels) that were used in all variant-based analysis. 

The intron/exon boundaries in *SfABCC2* were determined by alignment to a *SfABCC2* cDNA (GenBank accession number KY489760.1) using Exonerate with the est2genome model option [30]. To confirm the accuracy of the intron/exon boundaries, we performed the same alignment of the *SfABCC2* gene with three additional available cDNA sequences from Cry1F-susceptible *S. frugiperda* (GenBank accession numbers MG387043.1, MN399979.1 and KY646296.1). The same intron/exon boundaries were identified in each generic feature file (GFF) generated by Exonerate. The consensus protein sequence from aligning the SfABCC2 sequences derived from translating the *SfABCC2* cDNA sequences above in CLC Genomics Workbench 21.0.4 (Figure S2) was used as a reference to locate amino acid changes from mutations. This consensus SfABCC2 protein sequence differs in two amino acid positions in the notation for mutations located to extracellular loop 4 region presented by Boaventura et al. [10].

Functional annotation was performed with SnpEff [31], and SnpSift was used to filter annotations to a tabular format [32]. The final filtered set of 1333 variants contained 17 missense mutations and 1 nonsense mutation. Compared to the unfiltered variant set, the filtered set excluded one nonsense mutation because of both alternate allele frequency and read depth criteria, and two missense mutations because of the alternate allele frequency criterion. 

We also computed PCA projections via PLINK [33], and visualized them in ggplot2 [34]. Given the highly polymorphic nature of the samples, we also determined haplotypes for PCA and generated neighbor joining trees using the ShapeIT software [35]. Since no recombination maps are available for *S. frugiperda*, the recombination rate in ShapeIT was set to 0.000023 cM/Mb based on previous research for *Spodoptera litura* [36]. 

Admixture model-based clustering was performed using FastStructure [37] to identify genetic structure within the *SfABCC2* gene. FastStructure is a generative model-based approach based on Hardy–Weinberg equilibrium assumptions between alleles and linkage disequilibrium between genotyped loci. The FastStructure script structure.py was used to determine k, the number of assumed populations or genetic groups that share a subset of allele frequencies from *k* = 2 to 10. The choosek.py script in FastStructure chose *k* = 3 for model complexity maximizing the marginal likelihood, and *k* = 4 for model components to explain structure in the data. The final output of FastStructure was visualized using distruct.py at *k* = 4. 

## 3. Results

### 3.1. Genotyping Results Using Taqman

Based on Taqman genotyping, of the 41 *S. frugiperda* samples tested, 18 carried the *SfABCC2mut* allele, with nine being homozygous. Overall, these detections suggest an allele frequency of 0.3292 for *SfABCC2mut* in the analyzed samples, similar to previous estimations for the frequency of this allele in Puerto Rico [8]. The relative proportion of individuals carrying the *SfABCC2mut* allele at each location was higher in Lajas (53% carriers, including 33% homozygotes and 20% heterozygotes) followed by the Salinas (41% carriers, 8% homozygotes and 33% heterozygotes) and Santa Isabel (21% homozygotes) locations. 

### 3.2. Genotyping Results Using Targeted Sequencing

Overall, the multiplexed TILING primers amplified 63% of the *SfABCC2* reference gene from the *S. frugiperda* v.6.0 corn genome, with 23 of the 25 predicted exons located in contig 33 being fully sequenced (Figure S1). The Hi-Plex strategy does not rely on equilibration of primer efficiencies (or any kind of primer optimization) and as such, some targets will amplify more efficiently than others (or fail completely) and thus receive more sequencing (or none at all). To accommodate these expected variations in template concentration, the samples were sequenced to high depth (>100× per target amplicon). 

Of the 41 samples genotyped via targeted sequencing, 14 contain the *SfABCC2mut* GC insertion, with 8 scored as heterozygous and 6 as homozygous, resulting in a 0.2683 allele frequency. There was a 73% (30/41) concordance rate between the Taqman and the k-mer-based *SfABCC2mut* genotyping (Table 1). Five samples identified as susceptible by the k-mer-based genotyping were identified as heterozygotes (3) or homozygote resistant (2) by the Taqman assay. Furthermore, three heterozygous samples, based on k-mer-based genotyping, appeared as homozygous resistant in Taqman tests. Finally, two samples identified as homozygous resistant by the k-mer method were classified as homozygous susceptible by the Taqman assay. Examination of the assembled targeted sequences for the discrepant samples detected a number of SNPs in the *SfABCC2mut* site targeted by Taqman primers, which likely affected Taqman amplifications and therefore explain the observed discrepancies. In fact, of the 58 sites covered by the Taqman primers and probe, 19% were polymorphic in at least one of the 41 samples (data not shown).

### 3.3. Variant Analysis Pipeline and Genotyping

Functional annotation of variants detected using the bioinformatics pipeline shown in Figure 1 detected *SfABCC2mut* (2 bp GC insertion) as a frameshift mutation (D740A). There was a total of 18 samples in which the *SfABCC2mut* allele was detected using the GATK variant and filtering described earlier (see Methods). Within these 18 samples, 12 were heterozygous and 6 homozygous for the mutation, displaying 100% concordance with results from the k-mer-based genotyping approach. When a lower alternative allele threshold (≥3) was utilized, some discrepancies occurred between GATK and the k-mer-based genotyping (data not shown). 

In addition to identifying the known *SfABCC2mut* allele, we also detected 374 coding synonymous mutations (data not shown) and 23 nonsynonymous and frameshift mutations in *SfABCC2* in the filtered dataset. After mutations already observed in at least one *SfABCC2* cDNA from Cry1F-susceptible *S. frugiperda* were removed, 14 nonsynonymous and 9 frameshift mutations (apart from *SfABCC2mut* being synonymous with D740A) were identified as new potential Cry1F resistance alleles (Table 2 and Table S1). 

**Figure 2:**
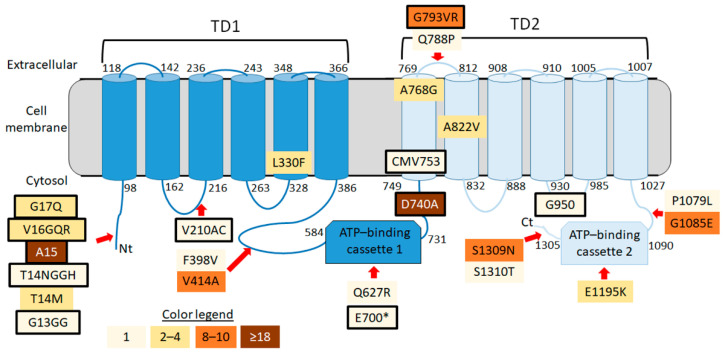
Schematic representation (not drawn to scale) of SfABCC2 topology on the cell membrane and location of detected nonsynonymous, nonsense and frameshift mutations in sequenced S. frugiperda samples from Puerto Rico. Amino acid numbers are indicated at the start and end of transmembrane domains and ATP-binding cassettes as predicted based on detection of conserved domains in BLAST searches. Distinct colors in boxes indicate the number of individuals carrying each mutation, as detailed in the color legend. The only nonsense mutation (E700 *) and all frameshift mutations are shown in a boxes with bold borders, including the *SfABCC2mut* allele (D740A). TD = transmembrane domain.

All missense (13) and nonsense (1) nonsynonymous mutations, except for an homozygous sample for T14M, were detected in heterozygous individuals. The most abundant missense mutations (10 individuals each, 24% of samples) were G1085E and S1209N, which occur in the C terminal intracellular region of SfABCC2 (Figure 2). The other common missense mutation was V414A (carried by 9 individuals, 22% of samples), which occurs in the loop connecting the first SfABCC2 transmembrane domain and ATP-binding cassette. Mutation P1079L was localized to a predicted splice site region. Only one missense mutation (Q788P, found in 1 sample) was localized to the fourth extracellular loop, a region hypothesized to be vital to Cry1F toxicity [10,11]. Missense mutations had a predicted moderate effect on the SfABCC2 protein, while the only nonsense mutation (E700 *) was predicted to result in a truncated SfABCC2 protein encompassing up to the first ATP-binding cassette (Figure 2).

Frameshifts were comparatively more frequent than nonsynonymous mutations, with homozygous individuals detected for all the frameshift mutations for which more than one positive sample was detected. These frameshift mutations generated aberrant proteins and predicted premature stop codons, leading to truncated SfABCC2 proteins, which in homozygous individuals would correspond to a Cry1F-resistant phenotype [8,12]. The A15 mutation was the most common (49% of individuals), with a slightly lower allele frequency (0.3170) than *SfABCC2mut* (synonymous with D740A mutation). Only three samples (PRL_168, PRL_170, PRL_171) from the same location (Lajas) were heterozygous for both A15 and D740A. These samples could be resistant to Cry1F based on complementation of these two alleles.

### 3.4. SfABCC2 Sequence Variation

**Figure 3:**
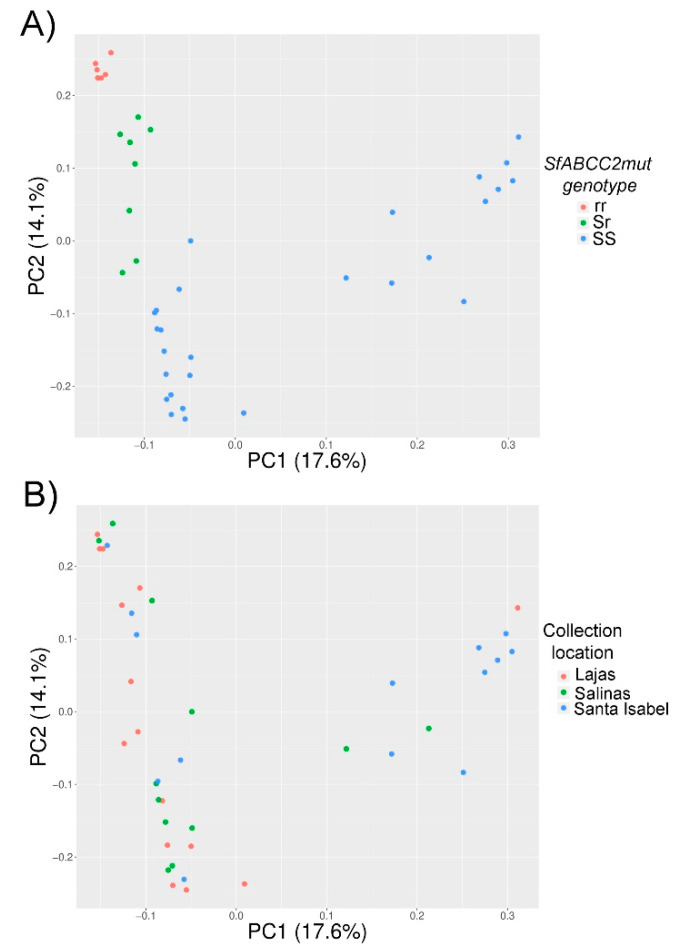
PCA projection plot of 1333 bi-allelic variants found within *SfABCC2* of 41 *S. frugiperda* samples and colored based on genotype for *SfABCC2mut* (**A**) and location (**B**). The *SfABCC2mut* genotype was determined as described in the Methods.

We also considered population-level diversity in *SfABCC2* sequences from Puerto Rico. Interestingly, a Principal Component Analysis (PCA) of all samples showed two distinct groups, one of them without *SfABCC2mut* in any individual (Figure 3A, right). Individuals homozygous for *SfABCC2mut* had the most compact cluster, suggesting more *SfABCC2* sequence variation within the SS compared to rr samples. 

**Figure 4:**
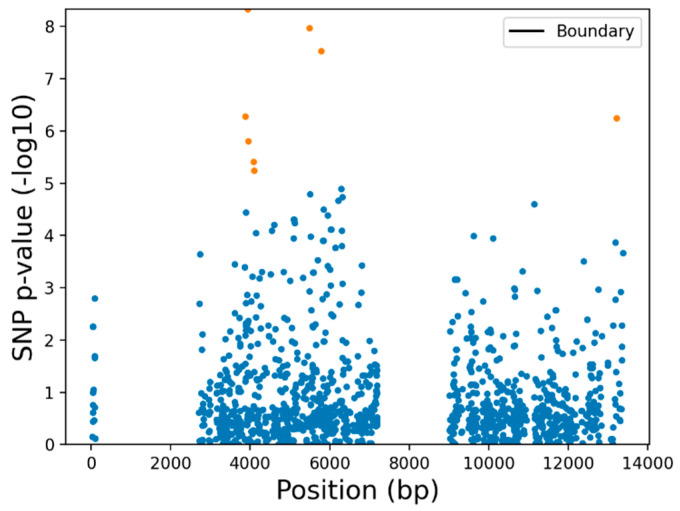
Manhattan plot of SNPs associated with PC2 (differentiating clusters of Sr and rr genotyped samples) on the filtered bi-allelic dataset (1333 variants).

To determine whether location was driving differentiation of the PC clusters observed given different frequencies of *SfABCC2mut* in the three Puerto Rico locations, we also plotted these samples colored by location (Figure 3B). No clusters correlated with sample location. Further, a logistic regression association test on PC1 vs. PC2 (Figure 4) generated eight SNPs with significant -log 10 *p*-value > 5 at positions 3874, 3936, 3944, 4085, 4097, 5485, 5788, and 13206. None of these variants, however, were predicted to have any functional effect change by SnpEff or corresponded to previously reported Cry1F-resistant alleles [8,10]. These data suggest that there may be barriers to Bt resistance gene flow, for example between host strains, even within the island of Puerto Rico.

**Figure 5:**
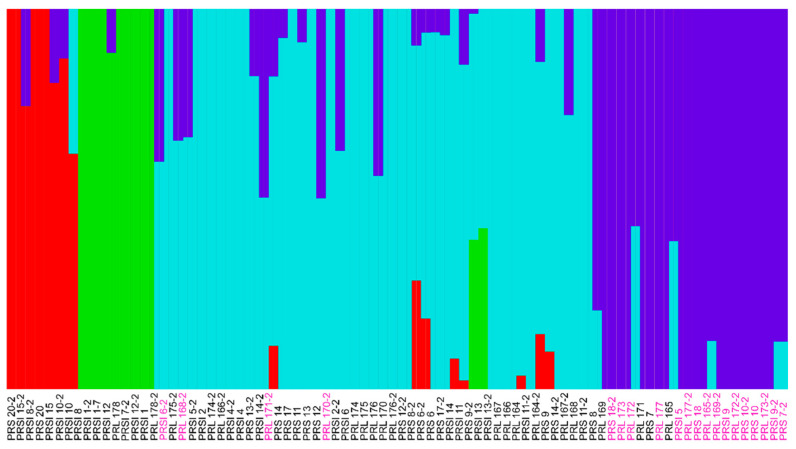
Genetic structure inferred by FastStructure on 82 haplotypes representing 41 samples at *k* = 4. Samples in pink text contain the *SfABCC2mut* allele.

To confirm this observation, we re-ran the PCA analysis without the *SfABCC2mut* homozygous individuals and observed the same clusters (data not shown). We next haplified our *SfABCC2* data using ShapeIt2 (1333 variants, see Methods) and ran FastStructure (Figure 5). Based on the targeted sequence data, we observed four populations, with the *SfABCC2mut* allele always associating with one population (purple color in Figure 5). Furthermore, even though there is little observed sharing of *SfABCC2* between the two primary PCA-based groups, admixture did occur between the *SfABCC2mut*-associated population and the three other populations. The frequency of the *SfABCC2mut* allele was highest in Lajas, indicating that this allele likely originated from this location and spread to other fall armyworm populations in Puerto Rico, as seen in the Structure plot. The observed moderate levels of admixture between the fall armyworms studied across all three Puerto Rican populations suggest that—contrary to the PCA results—the *SfABCC2mut* has the potential to spread to other subpopulations.

## 4. Discussion

Bioassay-based testing of F1 or F2 generations has traditionally been used to screen for resistance alleles in *S. frugiperda* to insecticidal proteins from *B. thuringiensis* produced in transgenic crops [5,14,38,39]. As an alternative, DNA-based screening methods provide higher sensitivity and are amenable to high-throughput applications and can considerably reduce overall labor and costs. Initial DNA-based resistance monitoring efforts for *S. frugiperda* focused on Taqman PCR targeting known mutations in the *SfABCC2* gene [8,9] and pooled population sequencing [10]. More recently, whole-genome resequencing was used to detect the presence of five reported resistance alleles in *S. frugiperda* populations from 12 geographic locations [40]. As a more cost-effective alternative to whole-genome sequencing, we provide proof of concept for the use of targeted highly multiplexed PCR for monitoring a known resistance allele (*SfABCC2mut*) and using bioinformatics to identify novel candidate *SfABCC2* resistance alleles. An advantage of the Cry1F-SfABCC2 system is that available knockout [12,13], mutational [11] and resistance [8,9] data in *S. frugiperda* support that a truncated SfABCC2 protein cannot function as Cry1F receptor. Thus, homozygote individuals for nonsense or frameshift mutations leading to truncated SfABCC2 proteins display a Cry1F-resistant phenotype, as observed for the *SfABCC2mut* resistance allele [8,9]. In addition, the amplification strategy of Hi-Plex allows the use of highly fragmented DNA, suggesting that poorly preserved samples, as is often the case for adult moths collected with pheromone traps, are amenable to analysis.

Both a k-mer-based and a bioinformatics pipeline provided the same genotyping results for a known mutation (*SfABCC2mut*). The genotypes of some samples differed between the Hi-Plex and Taqman methods. We observed that 19% of tested samples had polymorphisms around the priming/probe site for Taqman, which suggests an advantage of Hi-Plex over Taqman when dealing with highly polymorphic resistance genes. Most likely, the overlapping targets (and thus different priming sites) used in the TILING approach helps reduce the potential for erroneous genotypes and conclusions. 

We found that among the individuals carrying the *SfABCC2mut* allele, 14.6% were homozygotes, which are expected to produce a truncated SfABCC2 and thus be resistant to Cry1F [8,9,12,13]. The allele frequency of *SfABCC2mut* (0.3292) was in line with previous screening efforts in fall armyworm from Puerto Rico [8,9]. Based on the Faststructure and PCA-based analysis, no clear selective sweep was observed. The higher relative frequency of *SfABCC2mut* suggests the allele may have emerged in *S. frugiperda* in or around Lajas. Our population-level analysis suggests that the genomic region around *SfABCC2mut* is subject to both recombination and introgression, and that this occurs frequently, especially between populations at Santa Isabel and Lajas. In contrast, we still observed two main populations in Puerto Rico with statistically significant markers that do not associate with known or candidate resistance alleles. Further work is needed to determine whether there is at least one barrier to large-scale gene flow on the island, e.g., the well-established Corn vs. Rice strains in *S. frugiperda*. It is important to consider that Lajas has had rice winter nurseries (Univ. of Puerto Rico and Ricetech) for more than 30 years, while corn is the most prevalent crop at Santa Isabel. Furthermore, organic corn is planted in Lajas, which requires the frequent use of *B. thuringiensis* insecticides (e.g., Dipel) to control Lepidopteran pests [41].

In addition to *SfABCC2mut*, a second Cry1F-resistant allele was previously described in *S. frugiperda* from Puerto Rico [9]. In this case, insertion of a highly repetitive sequence at the beginning of the *SfABCC2* gene resulted in aberrant splicing and resistance. This type of allele would not be detected in our current Hi-Seq strategy as our TILING primers were not designed to amplify the highly repetitive sequence. Addition of primers amplifying this region and re-sequencing may allow detection and estimation of frequency for this allele. However, the reported highly repetitive nature of the insert [9] may hinder successful detection. 

Targeted sequencing combined with a custom bioinformatics pipeline allowed detecting, in addition to 374 coding synonymous mutations, 23 nonsynonymous and frameshift *SfABCC2* mutations as novel candidate Cry1F resistance alleles. A Cry1F-resistant phenotype similar to homozygous individuals for *SfABCC2mut* would be predicted for homozygous frameshift and nonsense mutations, as they would result in lack of a functional SfABCC2 protein [9,12,13]. Interestingly, some of the frameshift mutations were noticeably frequent. Importantly, a number of individuals positive for these frameshift mutations were homozygous samples predicted to produce truncated SfABCC2 proteins and thus having a Cry1F-resistant phenotype similar to *SfABCC2mut* [8]. In addition, heterozygous individuals could also be phenotypically resistant to Cry1F in cases of complementation between alleles involving nonsense and frameshift mutations. 

Significant gene flow between *S. frugiperda* populations from Brazil and Puerto Rico is not expected based on haplotype marker and meteorological observations [3]. In agreement with this observation, previous genotyping efforts did not detect *SfABCC2mut* among Brazilian *S. frugiperda* populations [9]. However, whole-genome comparisons suggest panmixia among *S. frugiperda* populations [42]. We did find two heterozygote individuals from Lajas (PRL_168 and PRL_164) carrying a GY deletion (DGY784D in our model, allele frequency 0.0571) previously described in Cry1F-resistant and field-collected *S. frugiperda* from Brazil [10]. However, detection of this GY deletion in Cry1F-suceptible individuals from Brazil [40] questions the relevance of this mutation to resistance. 

The extracellular loops 1 and 4 in SfABCC2 have been proposed as relevant to Cry1F toxicity [11], with loop 4 being most critical and also a hot spot for resistance mutations [10]. Only the Q788P mutation localized to that loop in our dataset. Interestingly, samples containing this mutation were detected in pooled sequencing of Brazilian *S. frugiperda* populations in one sample each from four locations (Balsas, Dourados, Santa Cruz das Palmeiras, and Uberlandia) [10]. These particular collection sites in Brazil are separated by > 1200 km, suggesting that the Q788P mutation may be common in Brazilian *S. frugiperda* populations. Among our samples, the mutation was rare and a single heterozygote individual (PRS_14) was detected. Functional tests would be needed to test the effect of Q788P on Cry1F toxicity.

Detection of common mutations may be suggestive of gene flow, including resistance alleles, between *S. frugiperda* populations from Brazil and the Caribbean. Although this flow is not expected based on predicted migratory movements [3], commercial exchange of contaminated plant materials could result in unintended dissemination of resistance alleles. This hypothesis should be further tested with increased sample sizes to better understand the potential paths for the spread of *S. frugiperda* resistance to *Bt* crops. 

Overall, our analyses detected 374 synonymous and 24 (including *SfABCC2mut*) nonsynonymous or frameshift *SfABCC2* mutations in 41 tested *S. frugiperda* individuals from Puerto Rico. These results indicate the *SfABCC2mut* and novel mutations predicted to result in truncated or aberrant SfABCC2 proteins and thus a Cry1F-resistant phenotype are not rare in *S. frugiperda* from Puerto Rico. Out of the 41 samples tested, the majority (38, 93%) carried at least one copy of a nonsense or frameshift *SfABCC2* mutation. There were 13 (32% of the total) homozygotes for these mutations, including 6 for *SfABCC2mut* and 6 for the A15 allele. Based on similar mutations in knockout and resistant strains of *S. frugiperda* [8,9,11,13], these homozygotes would display a Cry1F-resistant phenotype. The relatively high frequency of mutations predicted to result in resistance to Cry1F through SfABCC2 truncation is in line with the high levels of resistance to Cry1F maintained in Puerto Rico [18,43] and the lack of relevant fitness costs in disruptive *SfABCC2* mutants [15,44]. Our targeted approach does not allow us to identify additional Cry1F-resistant individuals carrying alternative resistance genes, which could potentially increase the frequency of Cry1F-resistant individuals. In addition, while missense *SfABCC2* mutations were also detected, a Cry1F-resistant phenotype cannot be predicted as their role in intoxication is unknown. For instance, the Q788P and A768G mutations could affect the predicted Cry1F-binding ability of the SfABCC2 loop 4 region [10]. Functional assays would be needed to test the effect of these mutations on susceptibility to Cry1F.

## 5. Conclusions

We provide proof of concept for the use of targeted sequencing in the monitoring of known mutations and the discovery of candidate resistance alleles to Cry1F in *S. frugiperda*. Furthermore, based on solid experimental evidence for the critical role of SfABCC2 in susceptibility to Cry1F, we predict the resistance phenotype of homozygous individuals for nonsense and frameshift mutations. This strategy provides higher sensitivity and should reduce labor and costs compared to traditional F1 and F2 screens to determine the frequency of diverse resistance alleles and, in specific mutations, susceptibility to Cry1F. Based on our observations, targeted sequencing overcomes the specificity limitations of Taqman genotyping, especially when targeting highly polymorphic genes. In addition, Taqman assays are limited to known alleles and cannot provide detection of novel candidate resistance alleles. Targeted sequencing provided preliminary evidence of a common mutation associated with Cry1F resistance in *S. frugiperda* from Brazil and Puerto Rico, which may suggest genetic flow between these populations as supported by whole genome-level comparisons [42]. Targeted sequencing such as Hi-Plex is adaptable to other pest-toxin resistance gene models, as long as known or suspected resistance loci are available, and the method can be expanded to sequence multiple genes within a single sample. While targeted sequencing provides an interesting list of candidate resistance alleles, further functional testing is needed to determine the role of missense mutations and their linkage with resistance to Cry1F. 

## Figures and Tables

**Table 1:** Concordance between results using Taqman and k-mer-based genotyping for the *SfABCC2mut* (GC insertion) allele, represented as the “r” allele. Samples with discrepant genotypes are highlighted in bold font.

Sample Name	Taqman	K-mer
PRL_164	SS	SS
**PRL_165**	**rr**	**Sr**
PRL_166	SS	SS
PRL_167	SS	SS
PRL_168	Sr	Sr
**PRL_169**	**rr**	**Sr**
PRL_170	Sr	Sr
PRL_171	Sr	Sr
PRL_172	rr	rr
PRL_173	rr	rr
PRL_174	SS	SS
PRL_175	SS	SS
PRL_176	SS	SS
PRL_177	rr	rr
**PRL_178**	**SS**	**SS**
**PRS_6**	**SS**	**SS**
PRS_7	Sr	Sr
PRS_8	SS	SS
**PRS_9**	**Sr**	**SS**
PRS_10	rr	rr
PRS_11	SS	SS
**PRS_12**	**Sr**	**SS**
PRS_13	SS	SS
PRS_14	SS	SS
**PRS_17**	**Sr**	**SS**
**PRS_18**	**SS**	**rr**
PRS_20	SS	SS
PRSI_1	SS	SS
PRSI_2	SS	SS
PRSI_4	SS	SS
**PRSI_5**	**SS**	**Sr**
**PRSI_6**	**rr**	**Sr**
**PRSI_7**	**rr**	**SS**
PRSI_8	SS	SS
**PRSI_9**	**SS**	**rr**
**PRSI_10**	**rr**	**SS**
PRSI_11	SS	SS
PRSI_12	SS	SS
PRSI_13	SS	SS
PRSI_14	SS	SS
PRSI_15	SS	SS

**Table 2:** Nonsynonymous and frameshift mutations identified by SnpEFF across 41 *S. frugiperda SfABCC2* bi-allelic variants. The nucleotide position in the *SfABCC2* gene reference (Position), the functional class of mutation (MISS = missense, NON = nonsense, and SHIFT = frameshift), the mutation with capitalized reference and alternative alleles, the corresponding amino acid change (Aa change), and the number of samples positive for that mutation (#Samples) are shown. Asterisks denote that the mutation leads to a premature stop codon. The *SfABCC2mut* allele is synonymous with D740A.

Position	Class	Mutation	Aa Change	#Samples
38	SHIFT	ggc/ggTGGTc	G13GG	1
40	SHIFT	acg/aATGGTGGTCATcg	T14NGGH *	1
41	MISS	aCg/aTg	T14M	4
42	SHIFT	gct/	A15 *	20
45	SHIFT	gtg/gGTCAACG	V16GQR *	4
48	SHIFT	ggc/CAggc	G17Q *	4
3421	SHIFT	gtc/gCATGtc	V210AC *	1
4077	MISS	Ctc/Ttc	L330F	2
4357	MISS	Ttt/Gtt	F398V	1
4540	MISS	gTg/gCg	V414A	9
6020	MISS	cAa/cGa	Q627R	1
6915	NON	Gaa/Taa	E700 *	1
7034	SHIFT	gat/gCGat	D740A *	18
7070	SHIFT	tgcatggtg/	CMV753	1
7120	MISS	gCt/gGt	A768G	3
9098	MISS	cAa/cCa	Q788P	1
9122	SHIFT	gga/GTAAgga	G793VR	8
9335	MISS	gCc/gTc	A822V	3
10491	SHIFT	gga/	G950	1
11708	MISS	cCg/cTg	P1079L	1
11726	MISS	gGa/gAa	G1085E	10
12507	MISS	Gaa/Aaa	E1195K	2
12748	MISS	aGt/aAt	S1209N	10
13285	MISS	Tcc/Acc	S1310T	1

## Data Availability

All targeted sequencing data used for this study are available through NCBI BioProject ID PRJNA729034.

## Supplementary Materials

The following are available online at https://www.mdpi.com/article/10.3390/insects12070618/s1, Figure S1: Representative partial visualization of targeted sequencing reads aligned to the *SfABCC2* reference gene; Figure S2: Consensus SfABCC2 protein sequence used in this study; Table S1: List of sequenced *S. frugiperda* samples and their genotype for detected mutations; Table S2: List of TILING primers used in PCR amplification of *SfABCC2*.
