# Supplementary material for: Genetic Screening to Identify Candidate Resistance Alleles to Cry1F Corn in Fall Armyworm Using Targeted Sequencing
Source: Insects. 2021 Jul 8;12(7):618. doi: 10.3390/insects12070618 (PMC8303720; doi:10.3390/insects12070618)
Supplement: Supplementary file 1 [file insects-12-00618-s001.zip › Supplementary Figures.pdf]

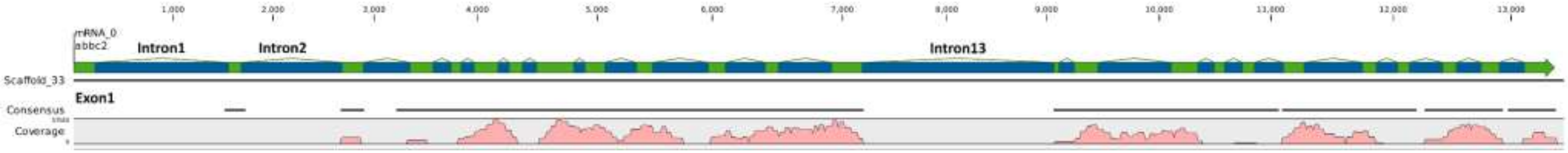

Figure S1- Representative overview (sample PRS_7 shown) of intron (blue) and exon (green) sequence coverage of the *SfABCC2* gene from the *S. frugiperda* corn v6.0 genome from LepidoDB when using overlapping TILING primers developed against an earlier gene model from genome assembly v3.1. Regions of the *SfABCC2* gene model with no sequence coverage are primarily intronic. The largest section with no coverage corresponds to the 1.9 Kbp of intron 13, which joined scaffolds 11087 and 7154 in the v3.1 genome version and was not available when the study was initiated. Other regions with no sequence coverage include exon 1 (218 bp), introns 1 and 2, and small sections (ranging from 32-322 bp) of introns 18, 21 and 23. In total, 23 of the 25 exons were covered by the TILING amplicons (~90%). This graphical overview was produced using CLC Genomics Workbench 9.5.3 with mapping at default parameters and sequence coverage was too deep to show the individual sequences (e.g. exon 2 had 19X coverage whereas other sites had >1,000X).

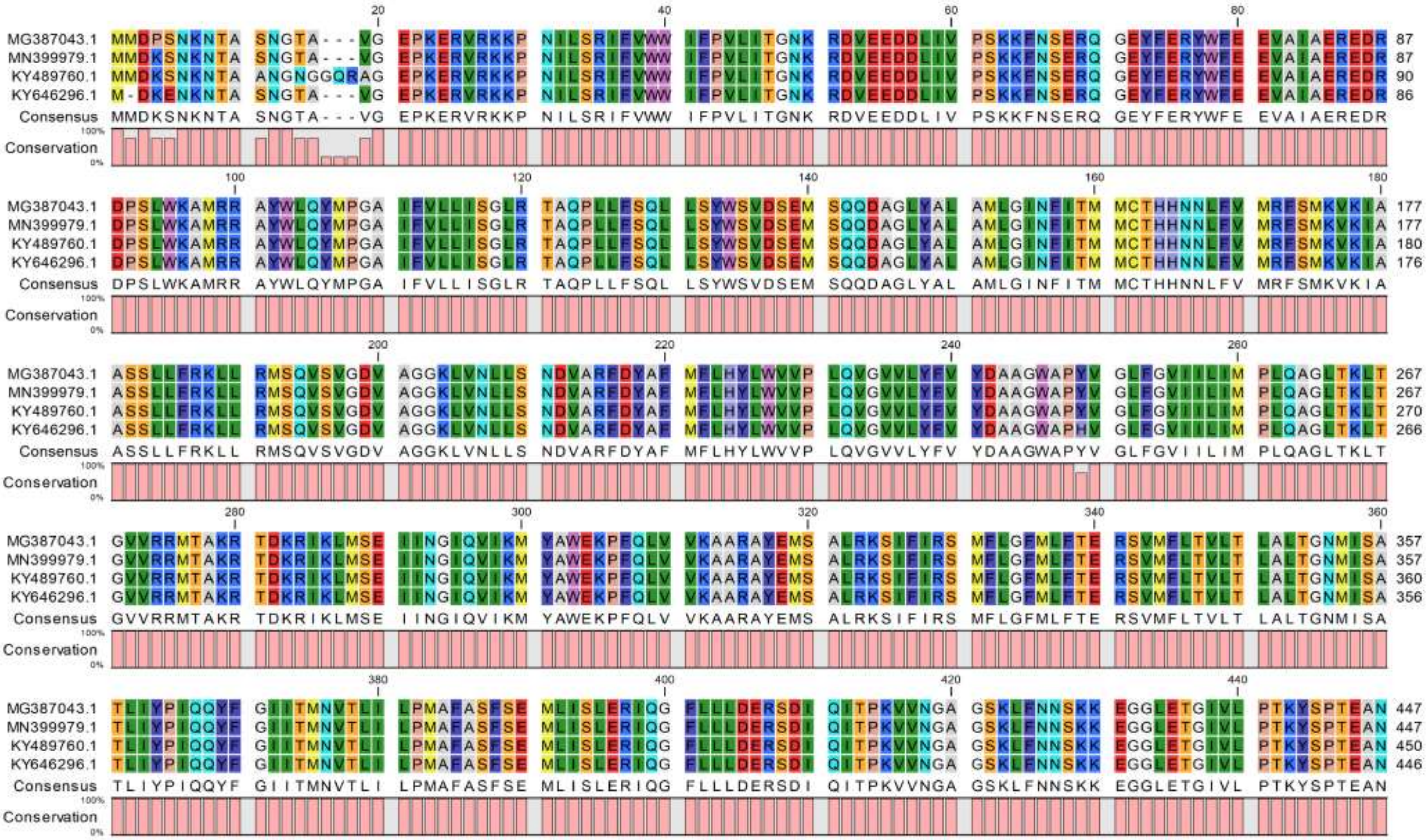

20 40 60 80
MG387043.1 MMDPSNKNTA SNGTA---VG EPKERVRKKP NILSRIFVWW IFPVLITGNK RDVEEDDLIV PSKKFNSERQ GEYFERYWFE EVAIAEREDR 87
MN399979.1 MMDKSNKNTA SNGTA---VG EPKERVRKKP NILSRIFVWW IFPVLITGNK RDVEEDDLIV PSKKFNSERQ GEYFERYWFE EVAIAEREDR 87
KY489760.1 MMDKSNKNTA ANGNGGQRAG EPKERVRKKP NILSRIFVWW IFPVLITGNK RDVEEDDLIV PSKKFNSERQ GEYFERYWFE EVAIAEREDR 90
KY646296.1 M-DKENKNTA SNGTA---VG EPKERVRKKP NILSRIFVWW IFPVLITGNK RDVEEDDLIV PSKKFNSERQ GEYFERYWFE EVAIAEREDR 86
Consensus MMDKSNKNTA SNGTA---VG EPKERVRKKP NILSRIFVWW IFPVLITGNK RDVEEDDLIV PSKKFNSERQ GEYFERYWFE EVAIAEREDR
Conservation 100% 0%
100 120 140 160 180
MG387043.1 DPSLWKAMRR AYWLQYMPGA IFVLLISGLR TAQPLLFSQL LSYWSVDSEM SQQDAGLYAL AMLGINFITM MCTHHNNLFV MRFSMKVKIA 177
MN399979.1 DPSLWKAMRR AYWLQYMPGA IFVLLISGLR TAQPLLFSQL LSYWSVDSEM SQQDAGLYAL AMLGINFITM MCTHHNNLFV MRFSMKVKIA 177
KY489760.1 DPSLWKAMRR AYWLQYMPGA IFVLLISGLR TAQPLLFSQL LSYWSVDSEM SQQDAGLYAL AMLGINFITM MCTHHNNLFV MRFSMKVKIA 180
KY646296.1 DPSLWKAMRR AYWLQYMPGA IFVLLISGLR TAQPLLFSQL LSYWSVDSEM SQQDAGLYAL AMLGINFITM MCTHHNNLFV MRFSMKVKIA 176
Consensus DPSLWKAMRR AYWLQYMPGA IFVLLISGLR TAQPLLFSQL LSYWSVDSEM SQQDAGLYAL AMLGINFITM MCTHHNNLFV MRFSMKVKIA
Conservation 100% 0%
200 220 240 260
MG387043.1 ASSLLFRKLL RMSQVSVGDV AGGKLVNLLS NDVARFDYAF MFLHYLWVVP LQVGVVLYFV YDAAGWAPYV GLFGVIILIM PLQAGLTKLT 267
MN399979.1 ASSLLFRKLL RMSQVSVGDV AGGKLVNLLS NDVARFDYAF MFLHYLWVVP LQVGVVLYFV YDAAGWAPYV GLFGVIILIM PLQAGLTKLT 267
KY489760.1 ASSLLFRKLL RMSQVSVGDV AGGKLVNLLS NDVARFDYAF MFLHYLWVVP LQVGVVLYFV YDAAGWAPYV GLFGVIILIM PLQAGLTKLT 270
KY646296.1 ASSLLFRKLL RMSQVSVGDV AGGKLVNLLS NDVARFDYAF MFLHYLWVVP LQVGVVLYFV YDAAGWAPHV GLFGVIILIM PLQAGLTKLT 266
Consensus ASSLLFRKLL RMSQVSVGDV AGGKLVNLLS NDVARFDYAF MFLHYLWVVP LQVGVVLYFV YDAAGWAPYV GLFGVIILIM PLQAGLTKLT
Conservation 100% 0%
280 300 320 340 360
MG387043.1 GVVRRMTAKR TDKRIKLMSE IINGIQVIKM YAWEKPFQLV VKAARAYEMS ALRKSIFIRS MFLGFMLFTE RSVMFLTVLT LALTGNMISA 357
MN399979.1 GVVRRMTAKR TDKRIKLMSE IINGIQVIKM YAWEKPFQLV VKAARAYEMS ALRKSIFIRS MFLGFMLFTE RSVMFLTVLT LALTGNMISA 357
KY489760.1 GVVRRMTAKR TDKRIKLMSE IINGIQVIKM YAWEKPFQLV VKAARAYEMS ALRKSIFIRS MFLGFMLFTE RSVMFLTVLT LALTGNMISA 360
KY646296.1 GVVRRMTAKR TDKRIKLMSE IINGIQVIKM YAWEKPFQLV VKAARAYEMS ALRKSIFIRS MFLGFMLFTE RSVMFLTVLT LALTGNMISA 356
Consensus GVVRRMTAKR TDKRIKLMSE IINGIQVIKM YAWEKPFQLV VKAARAYEMS ALRKSIFIRS MFLGFMLFTE RSVMFLTVLT LALTGNMISA
Conservation 100% 0%
380 400 420 440
MG387043.1 TLIYPIQQYF GIITMNVTLI LPMAFASFSE MLISLERIQG FLLLDERSDI QITPKVVNGA GSKLFNNSKK EGGLETGIVL PTKYSPTEAN 447
MN399979.1 TLIYPIQQYF GIITMNVTLI LPMAFASFSE MLISLERIQG FLLLDERSDI QITPKVVNGA GSKLFNNSKK EGGLETGIVL PTKYSPTEAN 447
KY489760.1 TLIYPIQQYF GIITMNVTLI LPMAFASFSE MLISLERIQG FLLLDERSDI QITPKVVNGA GSKLFNNSKK EGGLETGIVL PTKYSPTEAN 450
KY646296.1 TLIYPIQQYF GIITMNVTLI LPMAFASFSE MLISLERIQG FLLLDERSDI QITPKVVNGA GSKLFNNSKK EGGLETGIVL PTKYSPTEAN 446
Consensus TLIYPIQQYF GIITMNVTLI LPMAFASFSE MLISLERIQG FLLLDERSDI QITPKVVNGA GSKLFNNSKK EGGLETGIVL PTKYSPTEAN
Conservation 100% 0%

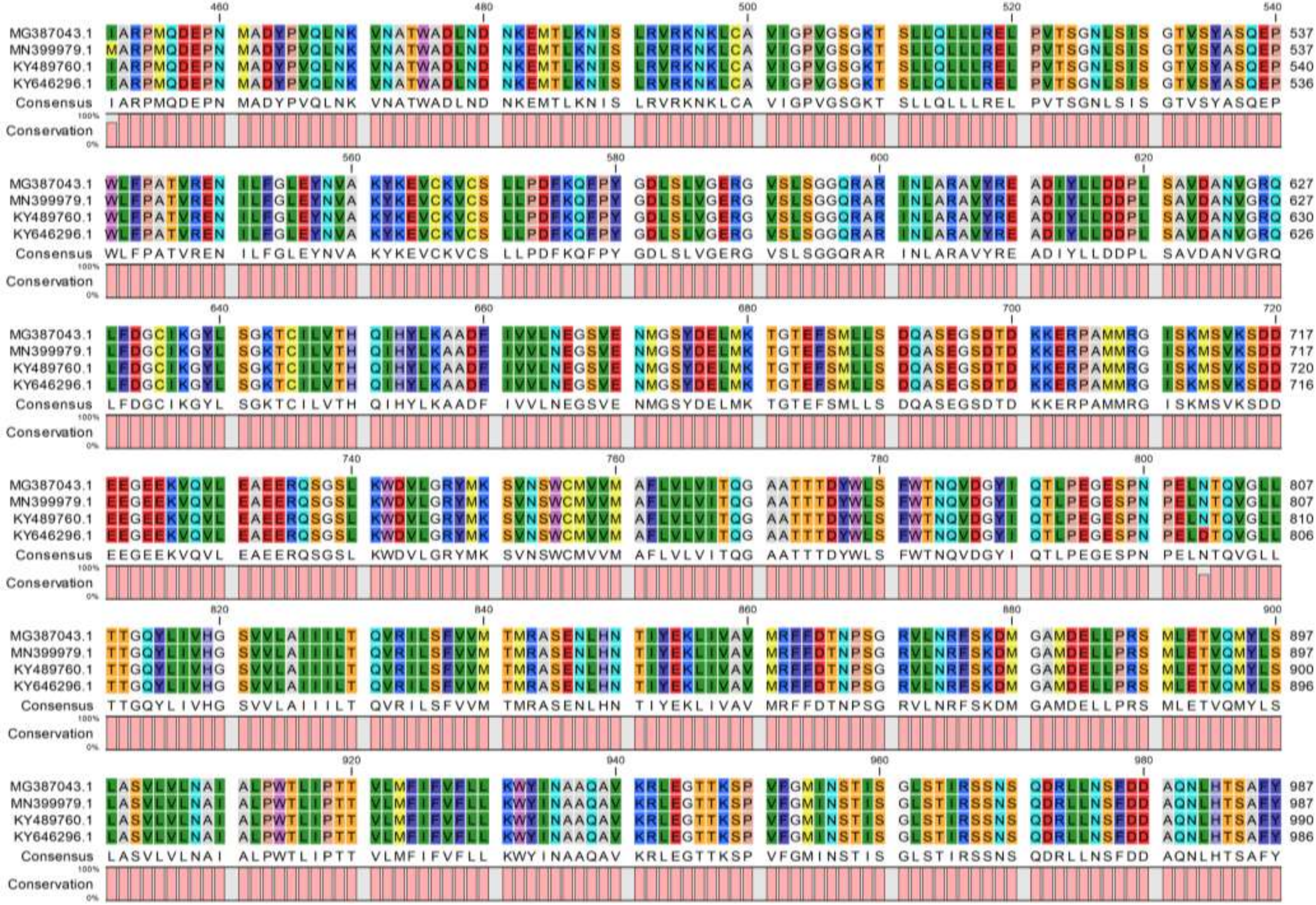
460 480 500 520 540
MG387043.1 IARPMQDEPN MADYPVQLNK VNATWADLND NKEMTLKNIS LRVRKNKLCA VIGPVGSGKT SLLQLLLREL PVTSGNLSIS GTVSYASQEP 537
MN399979.1 MARPMQDEPN MADYPVQLNK VNATWADLND NKEMTLKNIS LRVRKNKLCA VIGPVGSGKT SLLQLLLREL PVTSGNLSIS GTVSYASQEP 537
KY489760.1 IARPMQDEPN MADYPVQLNK VNATWADLND NKEMTLKNIS LRVRKNKLCA VIGPVGSGKT SLLQLLLREL PVTSGNLSIS GTVSYASQEP 540
KY646296.1 IARPMQDEPN MADYPVQLNK VNATWADLND NKEMTLKNIS LRVRKNKLCA VIGPVGSGKT SLLQLLLREL PVTSGNLSIS GTVSYASQEP 536
Consensus IARPMQDEPN MADYPVQLNK VNATWADLND NKEMTLKNIS LRVRKNKLCA VIGPVGSGKT SLLQLLLREL PVTSGNLSIS GTVSYASQEP
Conservation 100% 0%
560 580 600 620
MG387043.1 WLFPATVREN ILFGLEYNVA KYKEVCKVCS LLPDFKQFPY GDLSLVGERG VSLSGGQRAR INLARAVYRE ADIYLLDDPL SAVDANVGRQ 627
MN399979.1 WLFPATVREN ILFGLEYNVA KYKEVCKVCS LLPDFKQFPY GDLSLVGERG VSLSGGQRAR INLARAVYRE ADIYLLDDPL SAVDANVGRQ 627
KY489760.1 WLFPATVREN ILFGLEYNVA KYKEVCKVCS LLPDFKQFPY GDLSLVGERG VSLSGGQRAR INLARAVYRE ADIYLLDDPL SAVDANVGRQ 630
KY646296.1 WLFPATVREN ILFGLEYNVA KYKEVCKVCS LLPDFKQFPY GDLSLVGERG VSLSGGQRAR INLARAVYRE ADIYLLDDPL SAVDANVGRQ 626
Consensus WLFPATVREN ILFGLEYNVA KYKEVCKVCS LLPDFKQFPY GDLSLVGERG VSLSGGQRAR INLARAVYRE ADIYLLDDPL SAVDANVGRQ
Conservation 100% 0%
640 660 680 700 720
MG387043.1 LFDGCIKGYL SGKTCILVTH QIHYLKAADF IVVLNEGSVE NMGSYDELMK TGTEFSMLLS DQASEGSDTD KKERPAMMRG ISKMSVKSDD 717
MN399979.1 LFDGCIKGYL SGKTCILVTH QIHYLKAADF IVVLNEGSVE NMGSYDELMK TGTEFSMLLS DQASEGSDTD KKERPAMMRG ISKMSVKSDD 717
KY489760.1 LFDGCIKGYL SGKTCILVTH QIHYLKAADF IVVLNEGSVE NMGSYDELMK TGTEFSMLLS DQASEGSDTD KKERPAMMRG ISKMSVKSDD 720
KY646296.1 LFDGCIKGYL SGKTCILVTH QIHYLKAADF IVVLNEGSVE NMGSYDELMK TGTEFSMLLS DQASEGSDTD KKERPAMMRG ISKMSVKSDD 716
Consensus LFDGCIKGYL SGKTCILVTH QIHYLKAADF IVVLNEGSVE NMGSYDELMK TGTEFSMLLS DQASEGSDTD KKERPAMMRG ISKMSVKSDD
Conservation 100% 0%
740 760 780 800
MG387043.1 EEGEEKVQVL EAEERQSGSL KWDVLGRYMK SVNSWCMVVM AFLVLVITQG AATTTDYWLS FWTNQVDGYI QTLPEGESPN PELNTQVGLL 807
MN399979.1 EEGEEKVQVL EAEERQSGSL KWDVLGRYMK SVNSWCMVVM AFLVLVITQG AATTTDYWLS FWTNQVDGYI QTLPEGESPN PELNTQVGLL 807
KY489760.1 EEGEEKVQVL EAEERQSGSL KWDVLGRYMK SVNSWCMVVM AFLVLVITQG AATTTDYWLS FWTNQVDGYI QTLPEGESPN PELNTQVGLL 810
KY646296.1 EEGEEKVQVL EAEERQSGSL KWDVLGRYMK SVNSWCMVVM AFLVLVITQG AATTTDYWLS FWTNQVDGYI QTLPEGESPN PELDTQVGLL 806
Consensus EEGEEKVQVL EAEERQSGSL KWDVLGRYMK SVNSWCMVVM AFLVLVITQG AATTTDYWLS FWTNQVDGYI QTLPEGESPN PELNTQVGLL
Conservation 100% 0%
820 840 860 880 900
MG387043.1 TTGQYLIVHG SVVLAIIILT QVRILSFVVM TMRASENLHN TIYEKLIVAV MRFFDTNPSG RVLNRFSKDM GAMDELLPRS MLETVQMYLS 897
MN399979.1 TTGQYLIVHG SVVLAIIILT QVRILSFVVM TMRASENLHN TIYEKLIVAV MRFFDTNPSG RVLNRFSKDM GAMDELLPRS MLETVQMYLS 897
KY489760.1 TTGQYLIVHG SVVLAIIILT QVRILSFVVM TMRASENLHN TIYEKLIVAV MRFFDTNPSG RVLNRFSKDM GAMDELLPRS MLETVQMYLS 900
KY646296.1 TTGQYLIVHG SVVLAIIILT QVRILSFVVM TMRASENLHN TIYEKLIVAV MRFFDTNPSG RVLNRFSKDM GAMDELLPRS MLETVQMYLS 896
Consensus TTGQYLIVHG SVVLAIIILT QVRILSFVVM TMRASENLHN TIYEKLIVAV MRFFDTNPSG RVLNRFSKDM GAMDELLPRS MLETVQMYLS
Conservation 100% 0%
920 940 960 980
MG387043.1 LASVLVLNAI ALPWTLIPTT VLMFIFVFLL KWYINAAQAV KRLEGTTKSP VFGMINSTIS GLSTIRSSNS QDRLLNSFDD AQNLHTSAFY 987
MN399979.1 LASVLVLNAI ALPWTLIPTT VLMFIFVFLL KWYINAAQAV KRLEGTTKSP VFGMINSTIS GLSTIRSSNS QDRLLNSFDD AQNLHTSAFY 987
KY489760.1 LASVLVLNAI ALPWTLIPTT VLMFIFVFLL KWYINAAQAV KRLEGTTKSP VFGMINSTIS GLSTIRSSNS QDRLLNSFDD AQNLHTSAFY 990
KY646296.1 LASVLVLNAI ALPWTLIPTT VLMFIFVFLL KWYINAAQAV KRLEGTTKSP VFGMINSTIS GLSTIRSSNS QDRLLNSFDD AQNLHTSAFY 986
Consensus LASVLVLNAI ALPWTLIPTT VLMFIFVFLL KWYINAAQAV KRLEGTTKSP VFGMINSTIS GLSTIRSSNS QDRLLNSFDD AQNLHTSAFY
Conservation 100% 0%

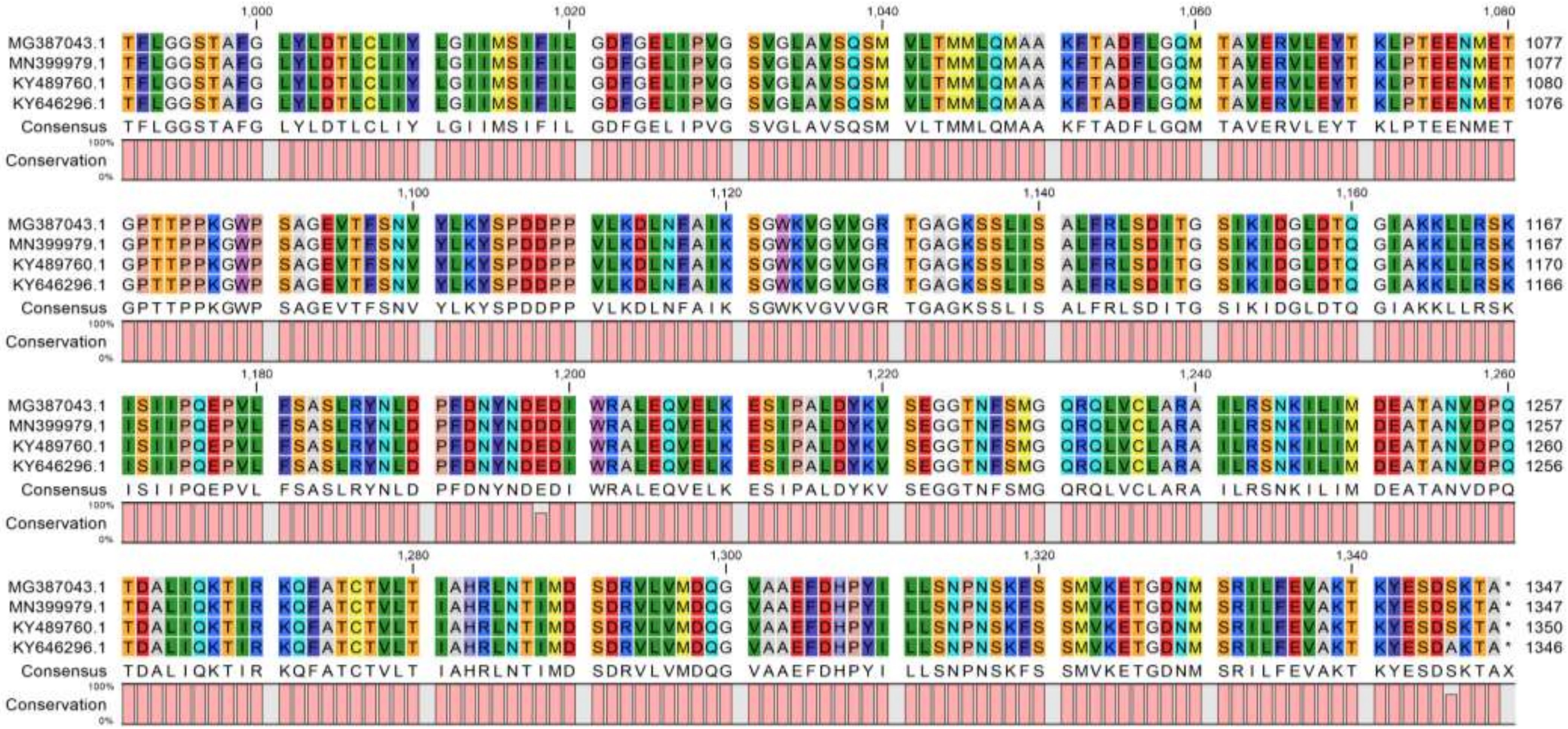

Figure S2- Alignment of *SfABCC2* cDNAs from NCBI (GenBank accession numbers MG387043.1, MN399979.1, KY646296.1 and KY646296.1) to obtain a consensus SfABCC2 protein sequence used as a reference to locate mutations. Sequence alignment and consensus determination was performed in CLC Genomics Workbench 21.0.4. Residue coloring represents Rasmol colors indicating traditional amino acid properties.
